# *Phoenix dactilyfera* L. Pits Extract Restored Bone Homeostasis in Glucocorticoid-Induced Osteoporotic Animal Model through the Antioxidant Effect and Wnt5a Non-Canonical Signaling

**DOI:** 10.3390/antiox11030508

**Published:** 2022-03-06

**Authors:** Samar R. Saleh, Doaa A. Ghareeb, Aliaa A. Masoud, Eman Sheta, Mohamed Nabil, Inas M. Masoud, Adham M. Maher

**Affiliations:** 1 Bio-Screening and Preclinical Trial Lab, Biochemistry Department, Faculty of Science, Alexandria University, Alexandria 21515, Egypt; daaa.ghareeb@alexu.edu.eg (D.A.G.); aliaa.ali@alexu.edu.eg (A.A.M.); adham.maher@alexu.edu.eg (A.M.M.); 2 Department of Pathology, Faculty of Medicine, Alexandria University, Alexandria 21515, Egypt; eman.sheta@alexmed.edu.eg; 3 Pharmacology Department, Faculty of Pharmacy, Deraya University, New Minya 61768, Egypt; mnm01120139193@gmail.com; 4 Pharmaceutical Chemistry Department, Faculty of Pharmacy, Pharos University in Alexandria, Alexandria 21515, Egypt; inas.masoud@pua.edu.eg

**Keywords:** date pits, oxidative stress, wnt signaling, RANKL/OPG ratio, DKK1and SOST, runx2

## Abstract

Oxidative stress associated with long-term glucocorticoids administration is a route through which secondary osteoporosis can be developed. The therapeutic potential of *Phoenix dactilyfera* L. pits is offered by their balanced, valuable and diverse phytochemical composition providing protective potential against oxidative reactions, making it a good candidate to treat glucocorticoid-induced osteoporosis (GIO). This study evaluates the possible anti-osteoporotic effect of date pit extract (DPE) against dexamethasone (DEXA)-induced osteoporosis. Male rats were allocated into three control groups, which received saline, low and high doses of DPE (150 and 300 mg/kg/day), respectively. Osteoporosis-induced groups that received DEXA (1 mg/kg/day) were divided into DEXA only, DPE (2 doses) + DEXA, and ipriflavone + DEXA. Femoral bone minerals density and bone mineral content, bone oxidative stress markers, Wnt signaling, osteoblast and osteoclast differentiation markers, and femur histopathology were evaluated. DPE defeated the oxidative stress, resulting in ameliorative changes in Wnt signaling. DPE significantly reduced the adipogenicity and abolished the osteoclastogenic markers (RANKL/OPG ratio, ACP, TRAP) while enhancing the osteogenic differentiation markers (Runx2, Osx, COL1A1, OCN). In Conclusion DPE restored the balanced proliferation and differentiation of osteoclasts and osteoblasts precursors. DPE can be considered a promising remedy for GIO, especially at a low dose that had more potency.

## 1. Introduction

For millennia, medicine has used plants as a source of therapeutic molecules. The first written records on medicinal applications of plants date to 2600 BC in Mesopotamia, containing about 1000 plant-derived medicines. “Ebers Papyrus” is an Egyptian medicinal record from about 1550 BC containing more than 700 drugs, mainly of plant origin [1]. Recently, plants have been under extensive investigation for biologically active compounds worldwide. The pits of *Phoenix dactilyfera* L. (Date palm) are a plant part that has not been adequately appreciated and is daily wasted in tons or utilized as animal feed for cattle, sheep, and camels [2]. Date pits (DP) have been investigated for pharmacological activities such as anti-inflammatory, immuno-stimulant, antidiabetic, antibacterial, antiviral, antioxidant, anti-tumorigenic, and anti-hypertension [3,4]. This is owing to their contents of phenols, minerals, amino acids, carotenoids, vitamins, tocopherols, phytosterols, and flavonoids [2,5].

Glucocorticoids (GC) are universally considered the drug of choice for treating inflammatory and autoimmune disorders. However, about 40% of patients on GC administration develop a fracture related to bone loss caused by the high GC dose and chronic treatment, which increases the risk of GC-induced osteoporosis (GIO) [6]. Bone remodeling results from the coordinated action of three distinct types of bone cells: osteocytes, osteoblasts, and osteoclasts. A variety of chemical agents, such as hormones, growth factors, and cytokines, also play a role in bone remodeling and homeostasis [7]. High doses of corticosteroids can trigger osteoporosis by distorting the balance between osteoclasts and osteoblasts through multiple mechanisms [8].

Oxidative stress occurs when the production of reactive oxygen/nitrogen species overrides the level of antioxidants, increasing the susceptibility for osteoporosis. GC can boost the generation of these reactive species, distorting bone remodeling and creating an imbalance between osteoclast and osteoblast, leading to osteoporosis [7,9]. Furthermore, oxidative stress can induce the apoptosis of osteoblasts and osteocytes while activating preosteoclasts differentiation. In addition, increased bone adiposity was also associated with GC administration [10]. Altogether favoring osteoclastogenesis over bone mineralization and osteogenesis [7]. Moreover, GC can affect bone indirectly, affecting calcium homeostasis by decreasing gastrointestinal calcium absorption and increasing renal calcium loss [11].

Wnt proteins are highly conserved glycoproteins that play a role in tissue development and homeostasis. Wnt proteins have been classified into canonical and non-canonical based upon downstream signaling effects (β-catenin dependent or independent, respectively) [12]. Dysfunctional Wnt signaling may contribute to the development of chronic metabolic diseases. Accounting for bone metabolic disorders and osteoporosis, the role of the canonical (Wnt/β-catenin) signaling pathway has been well established. The Wnt/β-catenin cascade is essential in maintaining bone mass balance as it controls numerous cell processes during early embryonic development and adult homeostasis. Wnt/β-catenin is critical for osteogenic differentiation of mesenchymal stem cells (MSCs), which can promote the transcription of osteoblast-related genes, such as Runt-related transcription factor 2 (Runx2), osterix (Osx), and collagen, type 1, alpha 1 (COL1A1). In case Wnt is not bound to its receptors, β-catenin is subsequently degraded by an active destruction complex composed of Axin, Adenomatous polyposis coli, casein kinase-1 and disheveled (Dsh), which promotes the phosphorylation of β-catenin depending on GSK-3β, resulting in ubiquitination and proteasomal degradation of β-catenin [13,14]. On the other hand, the binding of Wnt to Frizzled receptor (Fz) and LRP5/6 co-receptors promotes the recruitment of Dsh and the destruction complex to Fz, inhibiting the destruction complex, allowing the non-phosphorylated β-catenin to accumulate in the cytoplasm and translocate to the nucleus, activating Wnt target gene expression [15,16]. Aberrant Wnt/β-catenin signaling can lead to osteopenic states and skeletal disorders [13,16]. GC was found to produce severe alterations in Wnt/β-catenin pathway, disrupting the balance between osteoblastogenesis and adipogenesis of MSCs [13,17]. In contrast, the non- canonical Wnt proteins, Wnt5a, bind to the receptor complex of Fz and the receptor tyrosine kinase-like orphan receptors (Rors) 1/2 as co-receptors activating Wnt/Ca^2+^ pathways and Wnt/planar cell polarity pathways [18,19]. Compared to the canonical signaling, much less is known about the non-canonical one, in part may be due to its greater complexity. Certain kinds of literature try to investigate the crosstalk between Wnt signaling pathways. Interestingly, the non-canonical signaling can inhibit the canonical pathway at multiple levels and may affect the bone microarchitecture [12,14,20]. 

Regarding the beneficial effects of date pits extract (DPE), the present study was accomplished to appraise the possible anti-osteoporotic effect of two different doses of DPE against dexamethasone (DEXA)-induced osteoporosis in male rats through assessing the Wnt signaling pathway, osteoblasts’, and osteoclasts’ differentiation mediators and markers. Furthermore, ipriflavone (IPRI) is a synthetic isoflavone derivative and has commonly been used in the prevention and treatment of osteoporosis [21,22]. In this study, we used it as a reference drug.

## 2. Materials and Methods

### 2.1. Chemicals

Folin-Ciocalteau reagent, aluminum chloride, gallic acid, catechin, thiobarbituric acid, cumene H_2_O_2_, 5,5′-dithio-bis-2-nitrobenzoic acid (DTNB), p-nitrobenzyl chloride, reduced glutathione (GSH), pyrogallol, and DEXA were obtained from Sigma-Aldrich (St. Louis, MO, USA). Acid phosphatase (ACP), alkaline phosphatase (ALP), calcium, and total protein assay kits were purchased from Spectrum, Egypt. Osteocalcin (OCN), p38, peroxisome proliferator-activated receptors (PPARγ) and cluster differentiation-90 (CD90) ELISA kits were obtained from INOVA, China. Easy redTM total RNA extraction kit, as well as cDNA synthesis kit and RealMOD™ Green w2 2X qPCR mix kit, were purchased from iNtRON Biotechnology, Korea. Invitrogen, Thermo Fisher Scientific, USA was used to purchase the primers. Other chemicals were obtained with high grades.

### 2.2. Preparation of Date Pits Extract

DP extract was prepared as described in our previous published study [5]. Briefly, date pits were acquired from an Egyptian company in the Borg Al-Arab Industrial area, Alexandria, Egypt. Date pits were washed, left to dry, and finely grounded using a food grinder, then sieved to pass through 0.6 mm diameter. The powdered date pits were extracted twice after 72 h of maceration in 70% ethanol (200 g powdered date pits/1L 70% ethanol and reextracted in another 1L). The extract was then decanted, filtered, concentrated under reduced pressure, and vacuum freeze-dried to obtain a crude date pit extract (DPE, yield of 8%). 

### 2.3. Characterization of Date Pits Extract

Characterization of date pits extract was achieved by determining the antioxidant activity, measuring the total phenolic and total flavonoid contents, analyzing the phenolic acids profile using HPLC, and screening mineral content. 

The antioxidant activity of DPE was assessed using 1,1-diphenyl-2-picrylhydrazyl radical (DPPH) and hydrogen peroxide radical (H_2_O_2_)-scavenging activities [23]. For DPPH radical scavenging activity, 50 µL of different concentrations of DPE (dissolved in ethanol) was added to 100 µL of DPPH (0.2 mM, dissolved in ethanol). After a 30 min dark incubation, the absorbance was read at 490 nm against a blank that did not contain DPPH while the control does not include DPE. For H_2_O_2_ radical scavenging activity, 50 µL of different concentrations of DPE was mixed with 50 µL salicylic acid (9 mmol/L), 50 µL FeSO_4_ (9 mmol/L), and 50 µL of H_2_O_2_ (9 mmol/L). Then the mixture was incubated for 60 min at 37 °C, and the absorbance was measured at 510 nm. The control did not contain DPE, and the blank did not contain DPE and salicylic acid. The scavenging activity was expressed as IC_50_ (µg/mL).

Total phenolic content was estimated using the Folin-Ciocalteau colorimetric method described by Taga, Miller [24] using gallic acid as standard. Total flavonoids content was determined by aluminum chloride colorimetric method according to Zhishen, Mengcheng [25] using catechin as standard. Data were expressed as µg equivalent/mg extract. 

HPLC analysis of the phenolic compounds was conducted at Food Safety and Quality Control Laboratory (FSQC 0911-0915/2019), Faculty of Agriculture, Cairo University, Egypt. According to Lu, Yuan [19], HPLC analysis was performed using an Agilent 1260 Infinity HPLC Series (Agilent, Santa Clara, CA, USA), equipped with a quaternary pump. Phenolic substances were separated on a Kinetex 5 μm EVO C18 100 mm × 4.6 mm, (Phenomenex, Torrance, CA, USA) equipped with a variable-wavelength detector set at 284 nm. Retention time and peak spectra of standard phenolic compounds (gallic, rutin, catechin, protocatechuic, p-hydroxybenzoic, scopolrtin, chlorogenic, vanillic, caffeic, syringic, rosmarinic, p-coumaric, m-coumaric, ferulic, gentisic, hesperidin and naringin) were used for identification. All phenolic compounds were expressed as μg/g extract.

Mineral content (Ca, Cu, Fe, K, Mg, Mn, Na, P, Se, and Zn) of DPE was carried out by using atomic absorption spectroscopy (Agilent 5100 SVDV ICP-OES, U.S.) according to US EPA Method 200.7 [26] and US EPA Method 6010C [27]. One-gram DPE was digested in hydrochloric acid/nitric acid mixture (3:1) for 1 h at 100 °C and proceeded to be available for analysis. Data were expressed as µg/g extract.

### 2.4. Experimental Design

All animal procedures complied with the ARRIVE guidelines and were carried out following the Institutional Animal Care and Use Committee (IACUC protocol No.: AU-04 21 02 24 2 03, approved on 24 February 2021), Alexandria University, which fulfils the National Institutes of Health guide for the care and use of Laboratory animals (NIH Publications No. 8023). Fifty-six adult male Sprague Dawley rats weighing about 150–170 g were purchased from the animal house of the Graduate Studies and Research Institute, Alexandria University, Egypt. All rats were housed in polypropylene cages (4 animals/cage) and fed on a standard diet with ad libitum tap water. Rats were kept on a 12:12 h light-dark cycle in the well-aerated room under a controlled environment.

After one week of acclimatization, rats were allocated into seven groups (8 animals/group): Three control group received saline (Control), low-dose of DPE (LDPE, 150 mg/kg), and high-dose of DPE (HDPE, 300 mg/kg) daily for 8 weeks. One osteoporosis-induced group received dexamethasone (DEXA, 1 mg/kg, i.p.) daily for 6 weeks [28]. Three treated groups received two doses of DP (LDPE and HDPE) and IPRI (50 mg/kg) daily via gavage for 2 weeks before DEXA administration and they were continued for another 6 weeks with DEXA. The treated groups are illustrated as LDPE + DEXA, HDPE + DEXA, and IPRI + DEXA, respectively.

### 2.5. Collection and Preparation of Blood and Tissues

After nine weeks (the experimental period), animals were fasted overnight and were anaesthetized with isoflurane. Collected blood specimens were kept at room temperature for 15 min allowing it to clot, then centrifuged at 3000 rpm for 15 min at 4 °C to obtain serum that was stored at −20 °C for further use. 

The whole right-left femurs of all animals were collected immediately, then the muscles and the connective tissues were removed. For histopathological examination, four right femurs/group were fixed in 10% neutral formalin, whereas the other right femurs were used to assess bone mass. Washing of the left femurs with cold saline solution (0.9% NaCl) was followed by crashing under liquid nitrogen. The crushed femurs were divided into 2 parts kept at −80 °C. One part was taken to estimate the expression levels of the examined genes. The second part was homogenized using 9 volumes of cold phosphate buffer saline (0.1 M, pH 7.4), centrifuged at 4000 rpm for 15 min at 4 °C, and the supernatant was obtained and kept at −80 °C for biochemical estimation.

### 2.6. Bone Density Test

Femoral bone minerals density (BMD, g/cm^2^) and bone mineral content (BMC, g) were measured by dual-energy x-ray absorptiometry in Bone Minerals Density Unit, Medical Service Unit, National Research Center, Dokki, Egypt. The BMD was calculated as BMC/bone area.

### 2.7. Biochemical Analysis and Spectrophotometry Evaluations

Alkaline phosphatase (ALP) and acid phosphatase (ACP) activities were determined in rat sera spectrophotometrically using the manufacturer’s protocol of Spectrum company, Egypt. The lipid peroxidation marker, malondialdehyde (MDA), was estimated in bone homogenate supernatant following Tappel and Zalkin [29], which relies on thiobarbituric acid reactivity producing pink color adducts upon heating. The optical density was read at 532 nm, where bone MDA level was mentioned as µmol/mg protein. Griess reagent was used to determine bone nitric oxide (NO) level following the procedure described by Montgomery and Dymock [30]. The absorbance was recorded at 540 nm, and NO level was reported as µmol/g protein. Bone-reduced glutathione (GSH) level was evaluated following the method stated by Ellman [31]. GSH reacts with DTNB, generating a yellow-colored 2-nitro-5-thiobenzoic acid product. The absorbance of the developed color can be read at 412 nm. GSH level was expressed as µmol/mg protein. The bone glutathione peroxidase (GPx) activity was estimated using GSH, DTNB, and Cumene H2O2 as substrates. GSH reacts with DTNB forming a yellow-colored 2-nitro-5-thiobenzoic acid. The GPx activity can be determined by subtracting the excess GSH after enzymatic reaction from the total GSH in the absence of the enzyme. The absorbance was recorded at 412 nm, and GPx activity was expressed as U/mg protein [32,33]. Bone glutathione-S-transferase (GST) activity was assessed as designated by Habig, Pabst [34] using the substrates: p-nitrobenzyl chloride and GSH. GST catalyzes the development of glutathione nitrobenzyl. The absorbance was read at 310 nm, and GST activity was expressed as U/mg protein. Bone superoxide dismutase (SOD) activity was determined following Marklund and Marklund [35] using pyrogallol as substrate. One unit of SOD activity was defined as the amount of enzyme which inhibits the rate of pyrogallol auto-oxidation by 50%. SOD activity was expressed as U/mg protein. The total protein level in bone homogenate supernatant was measured according to the manufacturer’s protocol of Spectrum company, Egypt, to determine enzymes’ specific activities.

### 2.8. Sandwich ELISA

The levels of tartrate resistance acid phosphatase (TRAP), osteocalcin (OCN), peroxisome proliferator-activated receptors (PPARγ), cluster differentiation-90 (CD90), and p38 were determined in bone homogenate supernatant following the manufacturer’s instructions provided with Sandwich ELISA Kit (INOVA, Beijing, China). The levels of bone TRAP, CD90, and p38 were expressed in ng/mg protein, whereas OCN and PPARγ contents were expressed in pg/mg protein.

The levels of bone cluster differentiation-105 (CD105) was measured by the manual quantitative ELISA technique using rabbit polyclonal CD105 (#PA5-80582, Invitrogen, Waltham, MA, USA). In the polyvinyl chloride microtiter plate wells, the antigen was diluted to a final concentration of 100 µg protein in a coating buffer (carbonate buffer, 0.2 M, pH 9.6). Samples were loaded in duplicate and incubated for 2 h at room temperature, then overnight at 4 °C. Bovine serum albumin (5%) was used as a blocking solution and was incubated for 1 h at room temperature. Primary and secondary antibodies were diluted in blocking solution (2%) to reduce non-specific binding. The primary antibody was incubated for 1 h at room temperature then overnight at 4 °C. The plate was washed six times (10 min each) and incubated with alkaline phosphatase-conjugated secondary antibody (Goat anti-rabbit IgG, ALP, #A8025, Sigma-Aldrich, St. Louis, MO, USA) for 2 h at room temperature. Then ρ-nitrophenyl phosphate disodium salt (PNPP, ALP substrate) was added and incubated for 15 min. The reaction was terminated using sodium hydroxide (3 M). The developed color was measured at 450 nm on a plate reader (Saniafi Diagnostics Pasteur, Paris, France), followed by the construction of a standard curve for CD105. The level of bone CD105 was defined in ng/mg protein.

### 2.9. Total RNA Isolation and Quantitative Real-Time Reverse Transcription PCR Analysis (qRT-PCR)

qRT-PCR was performed, ensuing MIQE guidelines. An easy redTM total RNA extraction kit (iNtRON Biotechnology, Korea) was used for femoral total RNA isolation following the manufacturer’s protocol. The concentration and purity of RNA were estimated by using a NanoDrop 2000 spectrophotometer (Thermo Scientific, Waltham, MA, USA). The absorbance was measured at 260 and 280 nm. Samples with A260/280 ≥ 1.8 were used further. Isolated RNA (one µg) was reverse transcribed using Maxime RT PreMix kit (iNtRON Biotechnology, Korea) following the manufacturer’s instructions. The qRT-PCR reaction was amplified using cDNA as a template and glyceraldehyde-3-phosphate dehydrogenase (GAPDH) as a housekeeping gene. Primers sequences and conditions are shown in Table 1. The PCR mixture was prepared as follows: 10 µL of Taq qPCR Green Master Mix (Vivantis, Malaysia), 1 µL of forward primer, 1 µL of reverse primer and 1 µL of template cDNA were dispensed in PCR tubes (0.2 mL) then completed to 20 µL with nuclease-free distilled water. PCR was performed using the following thermal cycling conditions; initial denaturation at 95 °C for 2 min, 35–40 cycles of denaturation at 95 °C for 15 s, annealing as shown in Table 1 and extension at 60 °C for 30 s. qRT-PCR was performed using a CFX96™ Real-Time System (BIO-RAD, Hercules, CA, USA). The quantities critical threshold (Ct) of the target gene was normalized with quantities (Ct) of the house- keeping gene (GAPDH) by using the 2^−∆∆Ct^ method to calculate the fold change in target genes.

### 2.10. Histopathological Study

Right femurs of rats (*n* = 4 each group) were fixed in 10% neutral formaldehyde for 72 h, followed by decalcification in 10% buffered EDTA (pH 7.4) for 1 week. Femurs were dehydrated then embedded in paraffin in accordance with the standard protocol [43], after which they were cut into 4 μm sections using a rotary microtome (Leica RM2125 RTS, Germany) and stained with hematoxylin-eosin (H&E) staining for examination under a light microscope to assess histopathologic changes using an Olympus CX23 microscope. Morphological results were evaluated by two independent histopathologists. For trabecular bone quality, the metaphysis was evaluated. The scoring system was used following Bitto, Polito [44] and Khajuria, Disha [45], where score 0 is for normal structure, score 1 for partially reduced trabecular bone, score 2 for markedly reduced and score 3 for totally absent trabecular bone. The percentage of the trabecular bone area (TBA) was also assessed. Score 0 was estimated for 90–100% bone area, score 1 for 60–90%, score 2 for 30–60%, and score 3 was used for TBA less than 30%. Epiphyseal plate thickness was measured in 40× power. Four different measures were taken to calculate the mean. Osteoblasts rimming cortical bone were counted under one high power field. All those features were evaluated using a computerized image analysis program (Leica Application suite 4.12.0). Bone marrow was also evaluated for the presence or the absence of fat cells (necrobiosis). 

### 2.11. Statistical Analysis

Data were described as a mean ± SD. Differences within groups were analyzed statistically using one-way ANOVA using the LSD test, and *p* < 0.05 was considered for statistical significance. SPSS 16.0 (Chicago, IL, USA) was used for these analyses. Heat map analyses were obtained by the ClustVis web server (https://biit.cs.ut.ee/clustvis/ accessed on 6 February 2022) [46].

## 3. Results

### 3.1. Characterization of Date Pits Extract

The antioxidant activity, total phenolic and total flavonoid contents, phenolic acids profile using HPLC of DPE are adopted in Table 2. DPE has potent antioxidant activity as illustrated by the free radical scavenging activity with IC50 values of 44.38 ± 2.56 and 112.17 ± 5.11 µg/mL against H_2_O_2_ and DPPH radicals, respectively. DPE contained high amounts of phenolics and flavonoids (301.97 ± 5.16 and 5.10 ± 2.75 µg equivalent/mg extract, respectively). In addition, HPLC analysis indicated the presence of p-hydroxybenzoic acid, resveratrol, quinol, vanillic acid, benzoic acid, and syringic acid in the highest concentrations and other phenolic compounds in lower concentrations with total phenolic contents of 4180.86 ± 18.5 µg/g extract. The presence of these compounds increased the importance of DPE due to the potential antioxidant activity of these phenolic compounds responsible for facilitating the free radical scavenging activity.

Moreover, examining the mineral content of DPE reveals its abundance in essential elements such as calcium, potassium, magnesium, sodium, and phosphorous, as well as trace elements such as selenium, manganese, copper, zinc, and iron (Table 3), which are essential to a healthy diet and vital for human health.

### 3.2. BMD, BMC, and Serum Calcium Level

**Figure 1:**
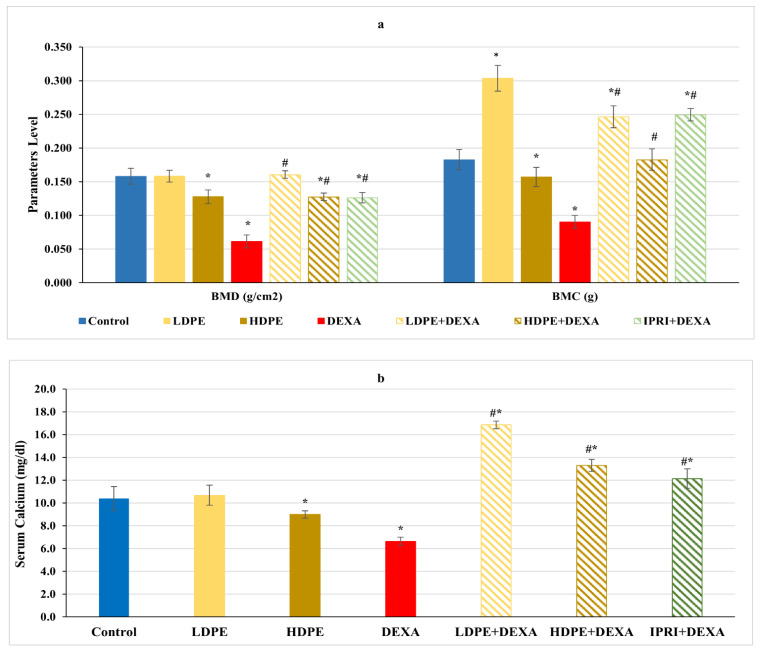
(**a**) Bone mineral density (BMD), bone mineral content (BMC) and (**b**) serum calcium level of the different experimental groups. Values are mean ± SD (*n* = 4 for BMD and BMC), (*n* = 8 for calcium level). *: significant (*p* < 0.05) compared to control. #: significant (*p* < 0.05) compared to DEXA. LDPE, low-dose of date pit extract (150 mg/kg); HDPE, high-dose of date pit extract (300 mg/kg); DEXA, dexamethasone (1 mg/kg); LPDE + DEXA, low-dose of date pit extract + dexamethasone; HPDE + DEXA, high-dose of date pit extract + dexamethasone and IPRI + DEXA, ipriflavone (50 mg/kg) + dexamethasone.

Concerning femoral bone minerals density (BMD), bone mineral content (BMC), and serum calcium level, rats injected with DEXA expressed a significant (*p* < 0.05) decrease in these parameters compared with the control rats (Figure 1). On the other hand, the treated groups received the low and the high dose of DPE (LDPE + DEXA and HDPE + DEXA), as well as the IPRI treated group (IPRI + DEXA), presented a significant (*p* < 0.05) increase in BMD, BMC, and serum calcium levels compared with DEXA-group. Interestingly, BMC and serum calcium levels in the three treated groups were significantly (*p* < 0.05) higher than in the control group. Moreover, the LDPE control group showed a non-significant change in BMC and serum calcium levels compared with the control rats and showed a better effect than HDPE (Figure 1).

### 3.3. Oxidative Stress Indices

Marked changes in the oxidative stress indices were noticed in DEXA rats’ femurs compared with the control rats (Table 4). Significant (*p* < 0.05) increases in MDA and NO levels were recorded, whereas a significant (*p* < 0.05) decrease in bone-GSH content along with a significant (*p* < 0.05) decline in bone GPx, GST, and SOD activities were reported in DEXA rats compared with the control rats. On the other hand, administration of either LDPE, HDPE, or IPRI to DEXA-rats caused significant (*p* < 0.05) decreases in MDA and NO levels associated with significant (*p* < 0.05) rises in GPx, GST, and SOD activities compared with DEXA rats. Interestingly, only LDPE and HDPE treatments caused a significant (*p* < 0.05) increase in GSH content compared with DEXA rats, and, significantly, (*p* < 0.05) increased above the control levels. In contrast, IPRI + DEXA rats showed significant (*p* < 0.05) differences in MDA, NO, and GSH levels compared with the control rats. Furthermore, LDPE + DEXA rats presented significant (*p* < 0.05) higher activities of GPx, GST, and SOD and elevated GSH level compared with control levels (Table 4).

### 3.4. Estimation of MSC Proliferation Markers

**Figure 2:**
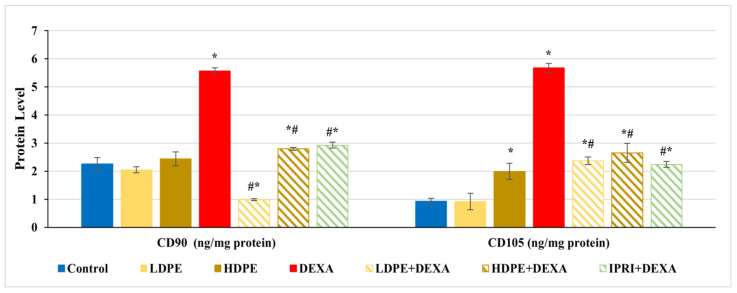
The protein levels of cluster differentiation-90 and -105 (CD90 and CD105) proliferation markers of the different experimental groups. Values are mean ± SD (*n* = 8). *: significant (*p* < 0.05) compared to control. #: significant (*p* < 0.05) compared to DEXA. LDPE, low-dose of date pit extract (150 mg/kg); HDPE, high-dose of date pit extract (300 mg/kg); DEXA, dexamethasone (1 mg/kg); LPDE + DEXA, low-dose of date pit extract + dexamethasone; HPDE + DEXA, high-dose of date pit extract + dexamethasone and IPRI + DEXA, ipriflavone (50 mg/kg) + dexamethasone.

Bone protein levels of cluster differentiation-90 and -105 (CD90 and CD105) were estimated as MSCs proliferation markers. CD90 and CD105 levels were significantly (*p* < 0.05) increased in rats injected with DEXA alone compared with control rats (Figure 2). Comparing these elevations with LDPE + DEXA, HDPE + DEXA, and IPRI + DEXA rats, a significant (*p* < 0.05) decrease in CD90 and CD105 levels was observed. However, their levels in the three treated groups remained significantly (*p* < 0.05) higher than the control rats, except the value of CD90 in LDPE + DEXA rats was significantly below the control level. Moreover, the administration of HDPE to control rats caused a significant (*p* < 0.05) increase in CD105 level, while the administration of LDPE to control rats showed non- significant changes compared with the control rats.

### 3.5. Osteoblast’s Differentiation Indices

**Figure 3:**
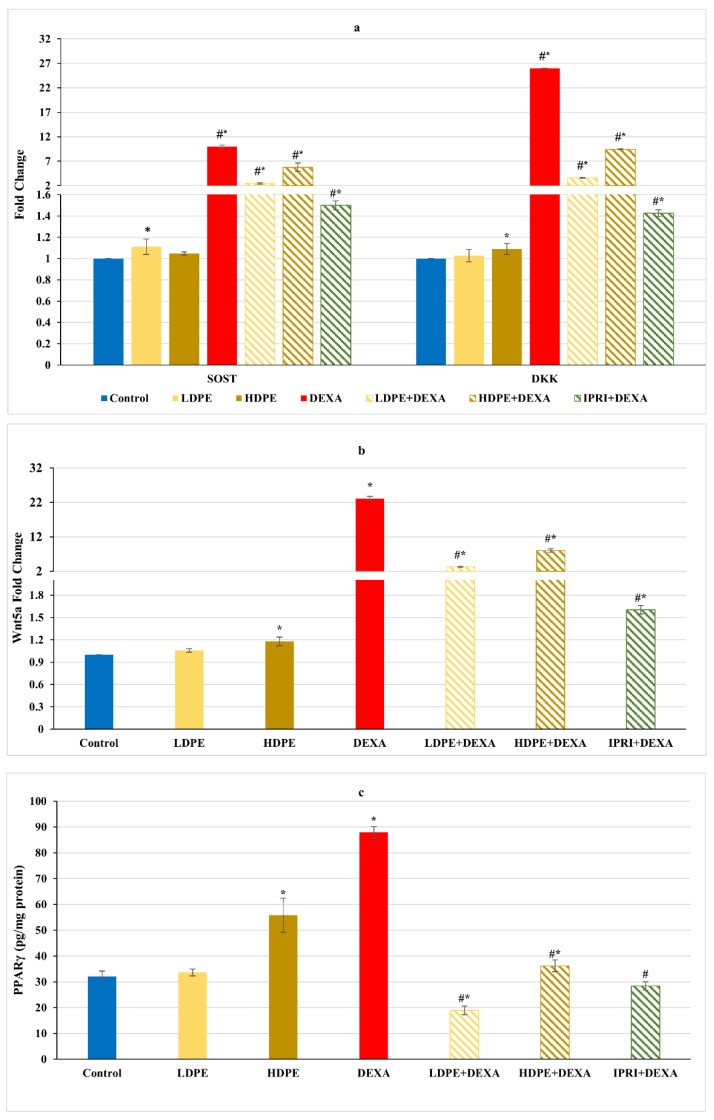
Osteoblast’s differentiation inhibitors of different experimental groups. (**a**) Bone sclerostin (SOST) and Dickkopf Wnt signaling pathway inhibitor 1 (DKK1) fold change. (**b**) Bone Wnt family member 5a (Wnt5a) fold change. (**c**) Bone protein content of peroxisome proliferator-activated receptors (PPARγ). Values are mean ± SD (*n* = 8). *: significant (*p* < 0.05) compared to control. #: significant (*p* < 0.05) compared to DEXA. LDPE, low-dose of date pit extract (150 mg/kg); HDPE, high-dose of date pit extract (300 mg/kg); DEXA, dexamethasone (1 mg/kg); LPDE + DEXA, low-dose of date pit extract + dexamethasone; HPDE + DEXA, high-dose of date pit extract + dexamethasone and IPRI + DEXA, ipriflavone (50 mg/kg) + dexamethasone.

Concerning Wnt/β-catenin signaling, the repeated injections with DEXA resulted in a significant (*p* < 0.05) increase in the fold change of Wnt/β-catenin inhibitors, including sclerostin (SOST), Dickkopf Wnt signaling pathway inhibitor 1 (DKK1), and Wnt family member 5a (Wnt5a), associated with a significant elevation in the master regulator of adipogenesis (PPARγ protein level) compared with the control group (Figure 3). These elevations were significantly (*p* < 0.05) reduced in DEXA rats administering either LDPE, HDPE, or IPRI. Administration of HDPE to control rats presented a significant (*p* < 0.05) increase in the expression level of DKK1 and Wnt5a as well as the protein level of PPARγ compared with the control group, while LDPE did not.

**Figure 4:**
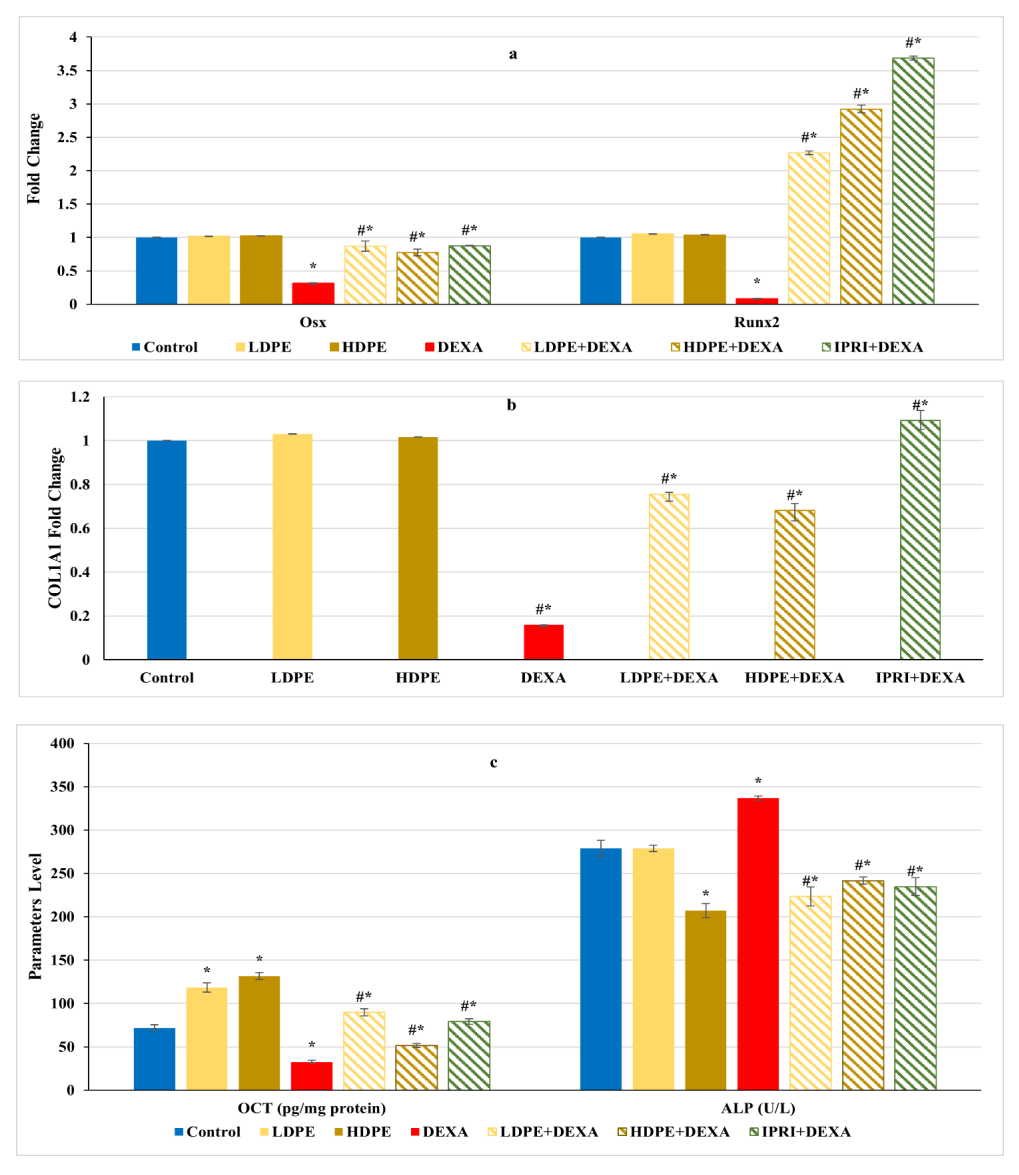
Osteoblasts’ differentiation markers of different experimental groups. (**a**) Bone Runt-related transcription factor 2 (Runx2) and osterix (Osx) expression. (**b**) Bone collagen, type 1, alpha 1 (COL1A1) fold change. (**c**) Bone osteocalcin (OCN) level and serum alkaline phosphatase (ALP) activity. Values are mean ± SD (*n* = 8). *: significant (*p* < 0.05) compared to control. #: significant (*p* < 0.05) compared to DEXA. LDPE, low-dose of date pit extract (150 mg/kg); HDPE, high-dose of date pit extract (300 mg/kg); DEXA, dexamethasone (1 mg/kg); LPDE + DEXA, low-dose of date pit extract + dexamethasone; HPDE + DEXA, high-dose of date pit extract + dexamethasone and IPRI + DEXA, ipriflavone (50 mg/kg) + dexamethasone.

On the other hand, the Wnt/β-catenin regulators and osteoblast-related genes, including Runx2, Osx, and COL1A1 gene expression levels, as well as the protein level of OCN, significantly (*p* < 0.05) declined after DEXA repeated injections compared with the control group (Figure 4a,b). Nonetheless, the administration of either LDPE, HDPE, or IPRI before and with DEXA resulted in a significant (*p* < 0.05) elevation in these parameters compared with the DEXA group. Interestingly, administration of LDPE to control rats showed a significant (*p* < 0.05) increase in OCN bone protein level and non-significant (*p* < 0.05) differences in the other parameters compared with the control group. 

On the other hand, DEXA injection significantly (*p* < 0.05) increased serum ALP activity compared with the control group (Figure 4c). However, all the treated rats (LDPE + DEXA, HDPE + DEXA, and IPRI + DEXA) exhibited a significant (*p* < 0.05) decrease in this activity compared with both DEXA-rats and control rats.

### 3.6. Osteoclast’s Differentiation Markers

**Figure 5:**
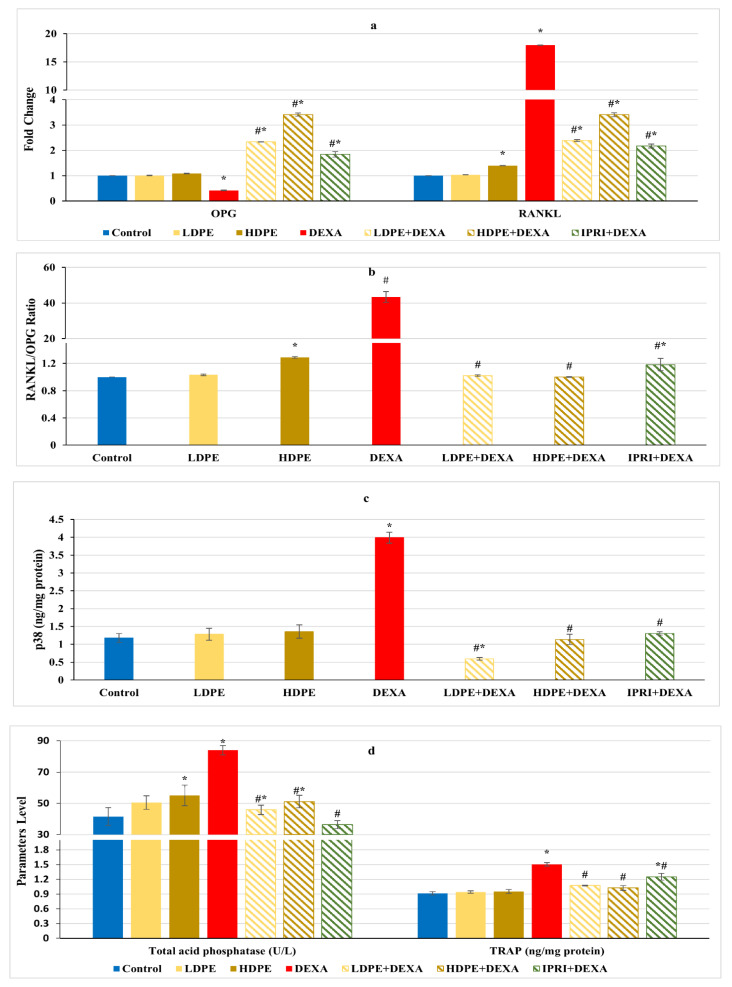
Osteoclast’s differentiation mediators and markers of different experimental groups. (**a**) Bone osteoprotegerin (OPG) and receptor activator for nuclear factor-kappa B ligand (RANKL) fold change. (**b**) Bone RANKL/OPG ratio. (**c**) Bone p38 protein content. (**d**) Serum acid phosphatase (ACP) activity and bone tartrate resistance acid phosphatase (TRAP) level. Values are mean ± SD (*n* = 8). *: significant (*p* < 0.05) compared to control. #: significant (*p* < 0.05) compared to DEXA. LDPE, low-dose of date pit extract (150 mg/kg); HDPE, high-dose of date pit extract (300 mg/kg); DEXA, dexamethasone (1 mg/kg); LPDE + DEXA, low-dose of date pit extract + dexamethasone; HPDE + DEXA, high-dose of date pit extract + dexamethasone and IPRI + DEXA, ipriflavone (50 mg/kg) + dexamethasone.

Regulation of RANKL/RANK/OPG signaling is essential for maintaining bone hemostasis. DEXA injected rats presented a significant (*p* < 0.05) decline in osteoprotegerin (OPG) fold change along with a significant (*p* < 0.05) increase in receptor activator for nuclear factor-kappa B ligand (RANKL) fold change as well as an amplified RANKL/OPG ratio compared with the control group (Figure 5a,b). These changes were significantly (*p* < 0.05) reverted following LDPE, HDPE, and IPRI administrations to DEXA-rats. OPG and RANKL fold changes, as well as RANKL/OPG ratio in the LDPE-control group, showed no significant (*p* < 0.05) differences from the control levels. 

In contrast with the control rats, the DEXA injected group triggered the release of bone turnover markers as indicated by a significant (*p* < 0.05) elevation in serum acid phosphatase (ACP) activity, bone tartrate resistance acid phosphatase (TRAP), and bone p38 protein content, compared with the control group (Figure 5c,d). Interestingly, LDPE + DEXA, HDPE + DEXA, and IPRI + DEXA rats experienced significant (*p* < 0.05) decreases in these parameters compared with DEXA rats. Moreover, the LDPE + DEXA group showed a significant (*p* < 0.05) decline in p38 protein content and non-significant (*p* < 0.05) changes in serum ACP activity compared with the control values (Figure 5c,d). 

### 3.7. Histopathologic Outcomes

**Figure 6:**
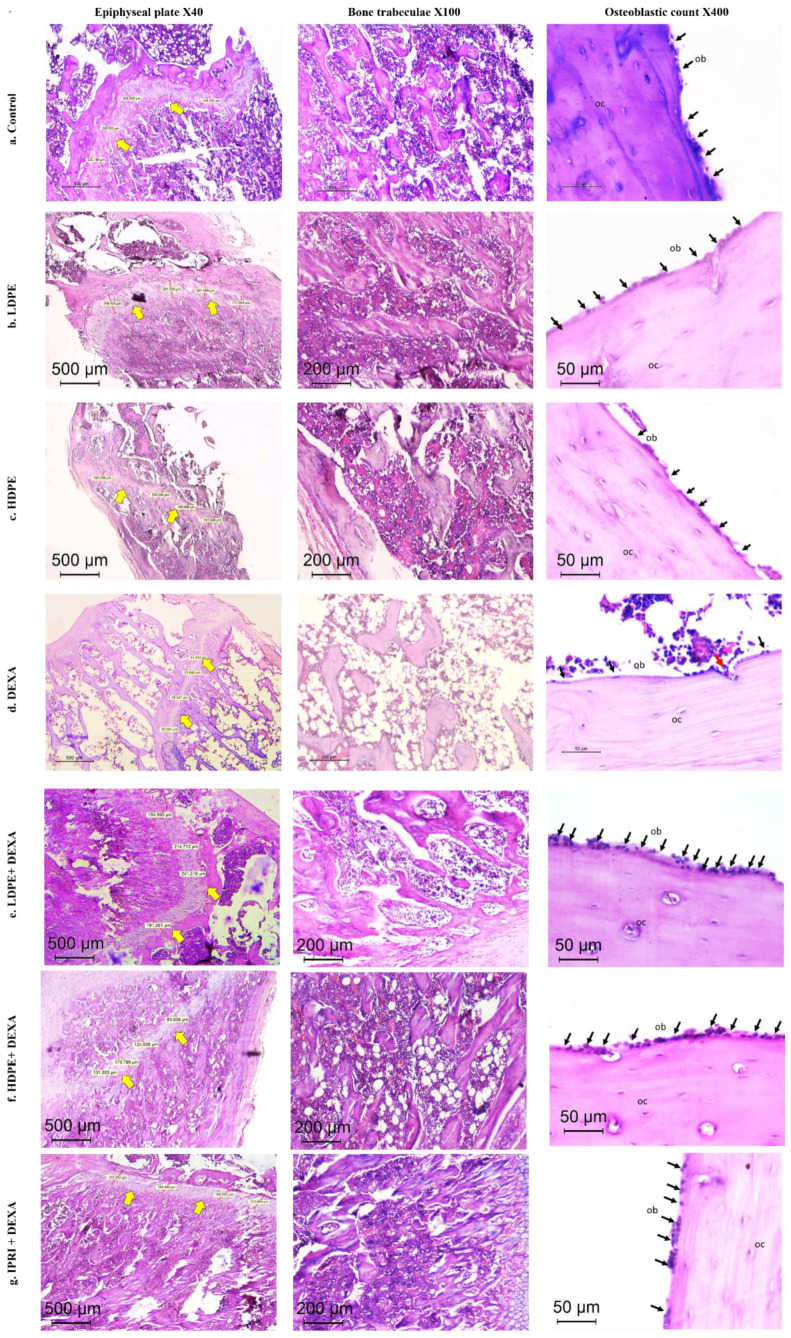
Histopathology of H&E-stained sections of the metaphysis of the femur in different studied groups. (**a**–**c**) Control, LDPE, and HDPE groups showed normal histology of epiphyseal plate, bone trabeculae, and osteoblastic rimming of cortical bone. (**d**) DEXA injected rats showed evident osteoporotic changes (thinned out the epiphyseal plate (×40), discontinuous thinned bony ossicles separated by the widened bone marrow, and increased fat content (×100), loss of osteoblastic rimming with increased osteoclastic activity with erosion cavity (×400)). (**e**–**g**) treated groups by different compounds showing reversal of osteoporotic changes in different degrees. Yellow arrows = epiphyseal plate. Black arrows (ob), osteoblasts; red arrow, osteoclasts; OC, osteocytes; LDPE, low-dose of date pit extract (150 mg/kg); HDPE, high-dose of date pit extract (300 mg/kg); DEXA, dexamethasone (1 mg/kg); LPDE + DEXA, low-dose of date pit extract + dexamethasone; HPDE + DEXA, high-dose of date pit extract + dexamethasone and IPRI + DEXA, ipriflavone (50 mg/kg) + dexamethasone.

The control group presented typical histology with well-ordered bone trabeculae arrangements, with an epiphyseal plate thickness of 277 µm and an osteoblastic mean count of 18/high power field (HPF). Thick and continuous bone trabeculae were observed (score 0), where trabecular bone areas (TBA) were scored 0. Both groups which received DP in low and high doses (LDPE and HDPE) showed similar findings with no pathologic changes detected (Table 5 and Figure 6). 

Additionally, DEXA-injected rats revealed induced osteoporosis acknowledged by the thinned epiphyseal plate of only 80 µm. Structural deterioration of bone trabeculae was also visible in the form of thinning, discontinuity, and irregularities in its architecture and was scored as 2 (markedly reduced). TBA was 0–30% (score 3). A sharp reduction in the osteoblast rimming bone cortex was noticed to be only 4/HPF with increased osteoclastic activity. Osteocytes were necrotic within their lacunae. Bone marrow space was widened with increased fat cells (Table 5 and Figure 6). 

DEXA rats receiving LDPE showed a marked recovery from the osteoporotic changes in histopathology. The epiphyseal plate mean thickness increased to 210 µm. The osteoblastic mean count increased to 18/HPF instead of 4/HPF in the DEXA model. The bone trabecular area increased to score 0, and trabecular bone quality was scored 1 compared to score 2 and 3 in the DEXA model, respectively. The bone marrow showed fewer fat cells. DEXA Rats receiving HDPE showed moderate prevention of DEXA-induced osteoporotic changes. The epiphyseal plate mean thickness measured 181 µm. The osteoblastic mean count was 13/HPF. The bone trabeculae were partially reduced (score 1), while the trabecular bone area ranged from 60–90% (score 1). Fat cells were still detected in the bone marrow space. The IPRI + DEXA treated rats showed improvement of osteoporosis; the epiphyseal plate thickness increased to 204 µm. The osteoblastic mean count increased to 15/HPF. The TBA increased to score 0, and trabecular bone was partially reduced to score 1. Interestingly, the improvement in DEXA rats administering LDPE (LDPE + DEXA) was slightly better than the group that received IPRI and much better than those receiving HDPE (Table 5 and Figure 6). 

### 3.8. Heat Map Analysis

**Figure 7:**
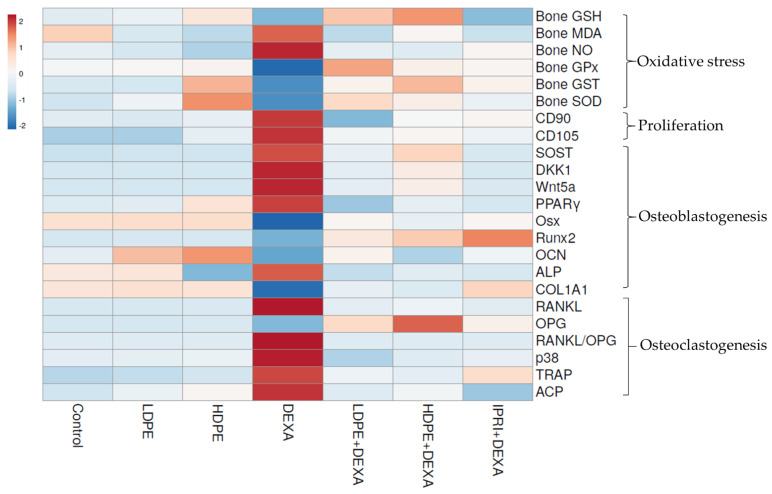
Heat map distribution of bone reduced glutathione (GSH), malondialdehyde (MDA), nitric oxide (NO), glutathione peroxidase (GPx), glutathione-S-transferase (GST), superoxide dismutase (SOD), differentiation-90 and -105 (CD90 and CD105), sclerostin (SOST), Dickkopf Wnt signaling pathway inhibitor 1 (DKK1), Wnt family member 5a (Wnt5a), peroxisome proliferator-activated receptors (PPARγ), osterix (Osx), Runt-related transcription factor 2 (Runx2), osteocalcin (OCN), collagen type 1 alpha 1 (COL1A1), receptor activator for nuclear factor-kappa B ligand (RANKL), osteoprotegerin (OPG), RANKL/OPG ratio, p38, tartrate resistance acid phosphatase (TRAP), serum acid phosphatase (ACP) and alkaline phosphatase (ALP). The color is distributed from blue (low level/expression or activity) to red (high level/expression or activity).

The examined parameters were hierarchically clustered by the heat map diagram (Figure 7) that indicated the correlation and the significance between the treated groups. The ClustVis tool was used to plot this diagram for clustering the multivariate data values. The chart’s color was related to the concentration of each parameter, where blue to red represents low to high level/expression or activity.

## 4. Discussion

Glucocorticoid-induced osteoporosis is characterized by a change in the population, function, and activity of the osteoclasts and osteoblasts, altering the bone remodeling process and affecting bone’s microarchitectural properties. GC can also indirectly affect bone by decreasing calcium availability [47]. BMD and BMC are considered as golden standard imaging tests for diagnosing osteoporosis [48]. The decline in BMD, BMC, and serum calcium levels experienced by DEXA-treated rats agrees with the findings of previous studies [40,49] and indicates incomplete bone mineralization. Furthermore, GC can trigger a declined serum calcium level by antagonizing vitamin D, decreasing intestinal calcium absorption, and decreasing renal calcium reabsorption [49,50]. All of which suggests the onset of osteoporosis.

Moreover, GC can trigger osteoporosis by inducing oxidative stress through depleting antioxidants, inhibiting antioxidant enzymes, and increasing lipid peroxidation [7]. In alliance with our outcomes, previous findings showed the association between GC administration and elevated MDA level and decreased bone GSH level, as well as the reduced activities of GST, GPx, SOD, and catalase [51]. Additionally, elevated NO can participate in the development of osteoporosis through prompting osteoblast apoptosis and enhancing osteoclast-mediated bone resorption, which is compatible with our findings [52,53]. On the other hand, GC can cause deficiencies in both testosterone and estrogen by decreasing the secretion of gonadotropin-releasing hormones and reducing the action of follicle-stimulating hormones [54]. These deficiencies contribute to the development of male osteoporosis owing to the central role of these sex hormones (mainly estrogen) in male bone homeostasis and maintaining a balanced reactive oxygen species (ROS) level through decreasing oxidative stress [55,56]. Moreover, oxidative stress can trigger the proliferation of hematopoietic stem cells (HSCs) and MSCs [57] while boosting osteoclast differentiation [9] and diverting MSCs differentiation towards the adipogenic lineage [58].

Wnt and RANKL/RANK/OPG signaling systems are two critical pathways regulated by osteoblasts that control bone mass by affecting bone formation and resorption through the regulation of osteoblasts and osteoclasts activities, respectively [18,59]. Osteoporosis is associated with the aberrant Wnt signaling pathways. Wnt signaling has been implicated as a critical regulator of bone repair and regeneration as well as adipose tissue development [15,60,61]. The majority of reports support the role of canonical Wnt signaling as anti-adipogenic while non-canonical Wnt signaling functioning as pro-adipogenic and promotes lipid accumulation [12,62]. Wnt/β-catenin controls MSC differentiation through activating the master transcriptional regulator Runx2 followed by Osx activation, which negatively regulates the master regulator of adipogenesis (PPARγ), retarding adipocyte differentiation [8,10,63,64]. In contrast, non-canonical Wnt signaling, perhaps through Wnt5a, has a direct effect on the adipose transcription factors by activating PPARγ and promoting adipogenesis [20]. Wnt5a is a multifactorial protein, and its action is related to which type of signaling pathways are activated. Wnt5a action was reported to be dose- and cell type-dependent; lower concentrations can prevent adipogenesis while higher concentrations are pro-adipogenic [20,65]. Furthermore, canonical and non-canonical Wnt signaling have intertwined roles in bone development, where the non-canonical signaling has been shown to inhibit the canonical signaling by multiple mechanisms. One such mechanism involves increasing the secretion of canonical Wnt antagonists/inhibitors (such as DKK1 and SOST) [12]. DKK1 and SOST inhibit Wnt signal transduction by binding to the LRP5 co-receptor, therefore blocking the interaction of the Wnt ligand to receptors, attenuating the transcription of Wnt osteogenic genes and genes of oxidant scavenging enzymes and accelerating differentiation and adipokine secretion in pre-adipocytes, promoting adipogenesis [66,67]. Moreover, SOST can stimulate RANKL secretion from osteocytes, increasing osteoclast differentiation [68]. Furthermore, increased DKK1 and SOST expression were reported to be associated with oxidative stress [66,69], implying a correlation between GC- induced oxidative stress and the inhibition of Wnt/β-catenin signaling pathway in these multipotent cells. In the present study, the increased expression of bone Wnt5a, DKK1, and SOST and elevated PPARγ protein content following DEXA injections indicated the activated non-canonical signaling, inhibited Wnt/β-catenin signaling and enhanced adipogenesis.

On the other hand, CD90 and CD105 are cell surface multipotency markers expressed by both MSCs, and HSCs [70,71]. These markers were measured in the current study, revealing an increase in their bone protein content after DEXA- repeated injections, suggesting the existence of proliferating MSCs and HSCs [72,73]. However, the commitment of MSCs may be towards either osteoblastic lineage or adipogenic lineage. Therefore, osteoblast’s differentiation markers were evaluated by measuring Runx2, Osx, OPG, COL1A1 and OCN. Runx2 and Osx are transcription factors that act downstream of the Wnt/β-catenin signaling in transcribing the bone-forming genes OPG, COL1A1, and OCN [74,75]. DEXA injections revealed a significant decrease in the expression levels of bone Runx2 and Osx and their downstream targets (COL1A1, OCN, and OPG), indicating a deactivated osteoblasts Wnt/β-catenin signaling.

Osteoclasts are multinucleated cells responsible for bone resorption. The osteoclasts differentiation is tightly regulated by osteoblasts and osteocytes. Osteoblasts and osteocytes would release RANKL and OPG, as well as macrophage colony-stimulating factor [18]. RANKL activates its cognate receptor (RANK) on the surface of osteoclasts and osteoclast precursors and stimulates preosteoclasts’ differentiation and adhesion to the bone matrix surface and promotes their activation and survival. OPG, the decoy receptor of RANKL, prevents RANKL-RANK binding and inhibits osteoclast differentiation [59]. Therefore, the modulation of RANKL/RANK/OPG signaling is one of the most crucial remodeling mechanisms during bone metabolism. The activation of the Wnt/β-catenin signaling in mature osteoblasts increases the production of OPG, which prevents bone resorption. In contrast, Wnt5a released by osteoblasts binds to Ror2 receptors and activates non-canonical signaling, promoting the expression of RANK and boosting RANKL-induced osteoclastogenesis and bone-resorbing ability by activating c-Jun N-terminal kinases [18,19,76]. Consistently, the increased bone RANKL expression and RANKL/OPG ratio in the DEXA-rats is a sign of increased bone resorption and bone loss due to the increased osteoclastogenesis [59,77]. In osteoclasts, the binding of RANKL to RANK leads to the activation of p38 resulting in the expression of TRAP and the consequent osteoclast differentiation. Furthermore, RANKL-induced osteoclast differentiation can promote ROS production, which further empowers the process of osteoclastogenesis [78]. We propose that the increased bone protein level of p38 can be related to the raised RANKL expression due to DEXA-induced oxidative stress. TRAP is an isoenzyme of ACP involved in bone resorption inside and outside the osteoclasts [79,80]. In alliance with our findings, Hozayen, El-Desouky [51] reported elevated serum ACP activity following DEXA- injections in female rats. This elevation was aligned with an increased bone TRAP, suggesting that ACP reflects the changes in bone TRAP. Furthermore, osteoclast differentiation was provoked by elevated Wnt5a signals through enhancing the expression of RANK in osteoclast precursors, thereby promoting RANKL-induced osteoclastogenesis. Therefore, Wnt5a, a typical non-canonical Wnt ligand, enhanced osteoclast formation [19,63,76]. 

Moreover, GC can induce apoptosis of osteoblasts through activating p38-MAPK [81]. In our study, the increased bone p38 protein content in the DEXA-induced group suggested increased osteoblast apoptosis as a result of oxidative stress and ROS accumulation in osteoblasts, as reported previously [82,83], and this explains the elevated serum ALP activity in DEXA-treated rats of this study, where osteoblast apoptosis promotes the release of osteoblasts’ content in the surroundings and finally rising serum ALP [84]. Interestingly, these findings are supported by the microscopic assessment showing the reduced number of osteoblasts, increased osteoclasts score, and increased femur adiposity, and introduce an actual model of osteoporosis following DEXA repeated injections. This can be explained by the GC-deleterious skeletal effects caused by the reduced bone matrix required for bone mineralization, activated bone resorption, and increased adipogenicity, in addition to decreased bone formation.

Ipriflavone, a synthetic isoflavone, is a nutraceutical used as a complementary, prophylactic, or alternative therapy for primary and menopause-related osteoporosis [85,86]. IPRI was found to prevent bone loss and enhance intestinal calcium absorption [87] and BMD [88]. In the present study, IPRI enhanced BMD, BMC, serum calcium level and maintained bone density as illustrated by the improved epiphyseal plate thickness, osteoblastic mean count and TBA compared with the DEXA-group. IPRI improves bone quality and protects bone tissue through direct and indirect actions that finally promotes MSCs osteogenesis. The anti-osteoporotic activity of IPRI is achieved through its ability to trigger osteoblast proliferation and maintain bone density while inhibiting bone resorption and retarding the osteoclasts multiplication [21,22]. An earlier study conducted by Yamazaki, Shino [89] reported the ability of IPRI to suppress bone resorption induced by the glucocorticoid prednisolone. Prednisolone reduced calcitonin secretion and increased blood parathyroid hormone levels, which led to increased bone resorption. IPRI modulated these parameters and suppressed bone resorption without affecting serum and bone calcium levels [89]. Recently, numerous studies have deduced that low doses of IPRI promotes the osteogenic differentiation of bone MSCs into osteoblasts by increasing ALP activity and osteoblast-specific gene expressions such as ALP, OCN, bone morphogenic protein 2, and OPG/RANKL ratio [21,88,90]. Our results are inconsistent with these findings where the administration of IPRI enhanced osteoblasts’ differentiation markers, including bone Runx2, Osx, COL1A1 expression levels and bone OCN level compared with the DEXA- induced group. Moreover, IRPI administration modulated the osteoclast’s differentiation mediators as indicated by the suppressed RANKL expression and RANKL/OPG ratio, bone p38 protein level and reduced bone turnover markers, TRAP protein level, and serum ACP activity. Moreover, the effect of IPRI on Wnt signaling is poorly studied. In the current study, we tried to investigate the anti-osteoporotic action of IPRI through Wnt signaling pathways. IPRI administration to an osteoporotic model (IPRI + DEXA) resulted in the suppression of the non-canonical Wnt signaling as indicated by the reduced Wnt5a, SOST, and DKK1 expression levels and decreased PPARγ protein level. These findings were also associated with a significant decline in MSCs proliferation parameters (CD90 and CD105). All together ensuring IPRI anti-adipogenicity function and the osteogenic differentiation of MSCs to a lesser extent than the low dose of DPE.

The antioxidant effect of IPRI is rarely studied, perhaps due to its poor solubility. In our previous study, we showed the ameliorative effects of IPRI upon oxidative stress markers (TBARS, NO, SOD, GPX, GST, and GSH) in rats exposed to environmental metal-induced neurodegeneration and dementia [91]. Solubility is an essential rate-limiting criterion for orally delivered medicines to achieve their required concentration in systemic circulation for pharmacological response [92]. Therefore, IPRI’s poor aqueous solubility limits its bioavailability and pharmacological actions [22]. Moreover, the use of IPRI is associated with various side effects such as headache, drowsiness, dizziness, depression, rash, and tachycardia [93]. Hence, the current study explored phytochemical compounds with more aqueous solubility and enhanced therapeutic potentials.

Medicinal plants are a good source of minerals and phytochemicals with protective effects against many inflammatory diseases along with the advantage of low toxicity and cost. The current study assessed the essential minerals in DPE, revealing generous concentrations. Minerals such as K, Ca, Mg, Fe, Mn, Cu, and Zn are essential to stabilize protein structure, break down peroxide and superoxide through electron transport (cytochrome), and activate antioxidant metalloenzymes as SOD (requires Cu, Zn, and Fe) [94], peroxidases and catalase (require Fe and Mg), as well as GPx (requires Se). All of these have a profound role in peroxide detoxification, defeating oxidative stress and preventing cell membrane damage [95,96], which can explain the regain of balanced bone antioxidant parameters.

Interestingly, Mg deficiency is associated with osteoporosis, where about 60% of total Mg is stored in the bone banked as an integral part of the apatite crystal. Intracellularly, Mg is essential for numerous physiological events such as stabilizing ATP and acting as a cofactor of enzymes involved in lipid, protein, and nucleic acid synthesis [97]. A considerable Mg concentration in DPE can partly restore the bone architecture in DEXA rats treated with low and high doses (LDPE + DEXA and HDPE + DEXA).

Potassium has been found to play an essential role in decreasing the risk of osteoporosis through maintaining acid-base balance, reducing the urinary loss of calcium through increasing calcium retention [98]. K/Na ratio is important for estimating the diuretic activity of medicinal plant extracts, where the ratio of diuretic drugs ranges from 5:1 to 615:1 [99]. K/Na ratio of DPE is about 6.6:1; this low ratio suggests a low diuretic activity, preserving the high calcium content and other minerals of DPE upon extract administration. In addition, the Ca/P ratio is vital for bone health. The adequate Ca/P ratio is (1–2:1), allowing ultimate availability and utilization of both minerals [100]. A decent Ca/P ratio of DPE about (1.3:1) is reported in this study and it is close to that of whole milk (1.27:1) [101]. This ratio reflects the balanced concentrations of both Ca and P, which is indispensable for a healthy bone. The rich mineral content of DPE can be partly in charge of the observed changes in BMD, BMC, and serum calcium levels following HDPE and LDPE administration.

During our previous published study [5], qualification and quantification of DPE were achieved and discussed, which revealed considerable quantities of fourteen phytochemicals raising the quality and importance of DPE, as these ingredients may oversee its free radical scavenging activity and antioxidant potential. Five out of the fourteen phenolics (p-hydroxybenzoic acid, resveratrol, quinol, vanillic acid, and benzoic acid) were found with considerable high concentrations in DPE. 

Polyphenols are the most widely distributed group of phytochemicals in the plant kingdom. Flavonoids are phenolic compounds that represent the most common and widely distributed group of plant phenolics [102]. Phenolic compounds possess an aromatic ring bearing one or more hydroxyl substituents. They act as antioxidants through free radical scavenging, metal chelation, upregulating or protecting antioxidant defenses, and suppressing ROS formation [103,104,105]. In agreement with our findings, previous studies reported polyphenols’ ability to revert oxidative stress in a GC-induced osteoporotic rat model [77,106].

Moreover, p-hydroxybenzoic acid, the highest constituent in DPE, was reported to have antioxidant properties against superoxide radicals [107]. Syringic acid [108], caffeic acid [77], and catechins [106] were reported to reduce oxidative stress in different rat models by decreasing MDA level while increasing the antioxidants, namely SOD, CAT, GPx activities, and GSH level, in bone tissue. Furthermore, resveratrol was reported to inhibit inducible nitric oxide synthase, reducing nitrosative stress in a rat periodontitis model [109]. Moreover, phytoestrogens are polyphenols with estrogen-like activity. They can regulate the expression of genes responsible for bone maintenance. Some of the phytochemicals found in DPE include p-hydroxybenzoic acid, catechins, vanillic acid, p-coumaric acid, ferulic acid, and resveratrol, exert estrogen-like activities that might be partly in charge of the anti-osteoporotic effects of DPE [110,111,112,113]. Hence, the high phenolic contents can be a major participant in DPE antioxidant activity against DEXA- induced oxidative stress, which explained the reduced levels of MDA, NO and p38 and the elevated GSH level as well as GPx, GST and SOD activities after DPE administration at low and high doses.

Previous studies support the current anti-osteoporotic actions of the active ingredients of DPE in various osteoporotic models. Interestingly, vanillic acid [113], resveratrol [114,115], coumaric, caffeic, and ferulic acids [77,116] were reported to stimulate cell proliferation and osteoblastic differentiation and increase the expression of osteoblastogenesis genes, including Runx2, COL1A1, Osx, OPG and OCN. Moreover, catechins were also able to reduce PPARγ expression in DEXA- induced osteoporotic mice [117], while resveratrol inhibited adipogenesis and enhanced osteoblastogenesis by restoring the Wnt/β-catenin pathway and suppressing PPARγ signaling [118]. Furthermore, vanillic and syringic acids [119], resveratrol [115,120], catechins [121], caffeic acid [77], and chlorogenic acid [122] were found to decrease the number of RANKL-induced osteoclastogenesis by reducing RANKL/OPG ratio supported by the reduced ACP and TRAP activities and/or gene expression level in osteoporotic rat models and cells. Moreover, chlorogenic [123] and ellagic acids [124] stimulated osteoblast precursors proliferation and differentiation in ovariectomized animal models and suppressed RANKL- induced osteoclastogenesis through suppressing p38-MAPK signaling pathways. Furthermore, rutin [125] and P-coumaric acid [126] improved bone histomorphometry and biochemical markers of bone turnover associated with reduced osteoclastic activity, increased bone mass/body mass ratio and bone mineral mass/body mass ratio in ovariectomized mice. 

Accordingly, we speculate that the changes following treating DEXA-induced osteoporotic rats with either LDPE or HDPE might be caused by direct or indirect actions of DPE polyphenols upon osteoporotic bone. The indirect actions are manifested in the antioxidant potential of phenolics within DPE, which can compete with DEXA- induced oxidative stress involved in interrupting Wnt signaling pathways. Furthermore, the rich mineral composition of DPE might aid in repairing the antioxidant system and provide the calcium necessary for bone formation. At the same time, the direct actions are aimed at the modifications triggered by DEXA upon Wnt signaling within and between bone cells through interacting directly with the components of Wnt pathways. 

The treatments caused a significant downregulation in CD90 and CD105 proliferation parameters, yet these parameters remained higher than control. This can be explained by the emergence of proliferating pre-osteoblasts, which can be supported by the increased osteogenic differentiation mediators (Runx2, Osx, OPG, and COL1A1) and markers (OCN) as well as the increased osteoblast score in the histopathological examination. Moreover, DPE suppressed the non-canonical signaling and reduced the adipogenic mediators (Wnt5a and PPARγ), decreased the osteogenic differentiation inhibitors (SOST and DKK1), ensuring the osteogenic fate of MSCs. The fact that SOST and DKK1 were still above the control values can be clarified by the need for osteogenic differentiation inhibitors to support the proliferating MSCs till they reach the osteogenic differentiation stage [67]. Furthermore, LDPE and HDPE caused a decline in osteoclastogenesis mediators (Wnt5a, RANKL, and RANKL/OPG ratio) and markers (ACP and TRAP) to be close to control levels, indicating re-balanced osteoclastogenesis. Finally, LDPE presented more profound amendments against the harmful effects of DEXA on bone than the HDPE and IPRI, making it the optimum dose against GIO. This can be related to the fact that polyphenols’ action can differ, depending on concentration and cellular developmental stage (cellular signaling context) [102].

The current study focused on Wnt5 non-canonical signaling and lacking the canonical (Wnt/β-catenin) signaling pathway. Therefore, further studies are required to investigate the role of DPE on the protein levels of bone β-catenin, glycogen synthase kinase 3β (GSK3β), phospho-GSK3β (GSK3βpS9) and Wnt co-receptor low-density lipoprotein receptor-related protein 5 (LRP5). Furthermore, the crosstalk between Wnt signaling pathways (canonical and non-canonical signaling) must be investigated.

## 5. Conclusions

DPE contains various phytochemical constituents and acts as minerals- rich nutrient that are essential for its potent radical scavenging ability and its powerful antioxidant capacity. Overall, the current data revealed the curative potential of low dose DPE against GIO earned by its ability to fight oxidative stress while intervening with Wnt signaling pathways. DPE significantly reduced the adipogenicity (reduced Wnt5a and PPARγ) and abolished the osteoclastogenic markers (diminished RANKL/OPG ratio, ACP and TRAP), while enhancing the osteogenic differentiation markers (elevated Runx2, Osx, COL1A1 and OCN) and finally restoring the Osteoblastogenesis/Osteoclastogenesis balance. The power of DPE to combat oxidative stress makes it a candidate worth exploring in other diseases.

## Figures and Tables

**Table 1:** Primers’ sequence and qRT-PCR conditions.

Gene Name	Accession Number	Primer Sequence		Annealing Temperature (°C)	Number of Cycles	Ref.
*GAPDH*	NM_017008.4	F	5′-AGATCCACAACGGATACATT-3′	52	35	[36]
		R	5′-TCCCTCAAGATTGTCAGCAA-3′			
*OPG*	NM_012870.2	F	5′-GTTCTTGCACAGCTTCACCA-3′	54	40	[37]
		R	5′-AAACAGCCCAGTGACCATTC-3′			
*RANKL*	NM_057149.1	F	5′-ACCAGCATCAAAATCCCAAG-3′	52	35	[37]
		R	5′-GGCCGCTAATTTCCTCACCA-3′			
*DKK1*	NM_001106350.1	F	5′-GCTGCATGAGGCACGCTAT-3′	55	35	[38]
		R	5′-AGGGCATGCATATTCCGTTT-3′			
*Wnt5a*	NM_022631.3	F	5′-CCATGAAGAAGCCCATTGGAATA-3′	60	40	[39]
		R	5′-GGCCAAAGCCATTAGGAAGAA-3′			
*SOST*	NM_030584.2	F	5′-GTGCAAGTGCAAGCGCCTCA-3′	60	40	[40]
		R	5′-GCTCCGCCTGGTTGGCTTTG-3′			
*Runx2*	NM_001278483.1	F	5′-AGTGTGTGTGTCCGCATGAT-3′	56	40	[41]
		R	5′-CCACTTGGGGTCTAAGAACG-3′			
*Osx*	NM_181374.2	F	5′-TGAGGAAGAAGCCCATTCAC-3′	53.5	40	[42]
		R	5′-ACTTCTTCTCCCGGGTGTG-3′			
*COL1A1*	NM_053304.1	F	5′-CAAGGACTATGAAGTTGATGC-3′	43	40	[40]
		R	5′-ACCAGTAGAGAAATCGCAGT-3′			

GAPDH, glyceraldehyde phosphate dehydrogenase; OPG, osteoprotegerin; RANKL, receptor activator for nuclear factor-kappa B ligand; DKK1, Dickkopf Wnt signaling pathway inhibitor 1; Wnt5a, Wnt family member 5a; SOST, sclerostin; Runx2, Runt-related transcription factor 2; Osx, osterix and COL1A1, collagen, type 1, alpha 1.

**Table 2:** Scavenging activity, Phytochemical content, and Phenolic compounds of DPE using HPLC analysis.

Scavenging Activity (IC_50_, µg/mL)			
**DPPH**		**112.17 ± 5.11**	
**H_2_O_2_ radical**		**44.38 ± 2.56**	
**Constituents**		**Concentration**	
**Phytochemicals**	**µg equivalent/** **mg extract**	**LDPE** **(150 mg/kg)**	**HDPE** **(300 mg/kg)**
**Total phenolics**	301.97 ± 5.16	45,295 ± 774	90,591 ± 1548
**Total flavonoids**	5.10 ± 2.75	765 ± 412.5	1530 ± 825
**HPLC Analysis of Phenolic Compounds**			
**Constituents**	**(μg/g extract)**	**LDPE** **(μg/150 mg/kg)**	**HDPE** **(μg/300 mg/kg)**
**Quinol**	**703.81 ± 8.5**	**105.6 ± 1.3**	**211.14 ± 2.55**
**p-Hydroxybenzoic acid**	**1042.01 ± 15.9**	**156.3 ± 2.25**	**312.6 ± 4.77**
**Catechin**	70.76 ± 2.0	10.61 ± 0.3	21.23 ± 0.6
**Chlorogenic**	26.67 ± 1.5	4.0 ± 0.225	8.0 ± 0.45
**Vanillic** **acid**	**625.43 ± 11.6**	**93.81 ± 1.74**	**187.63 ± 3.48**
**Caffeic acid**	89.58 ± 7.0	13.44 ± 1.05	26.87 ± 2.1
**Syringic acid**	**119.03 ± 3.9**	**17.85 ± 0.59**	**35.71 ± 1.17**
**p-Coumaric acid**	19.24 ± 1.9	2.89 ± 0.29	5.77 ± 0.57
**Benzoic acid**	**587.45 ± 17.6**	**88.12 ± 2.64**	**176.24 ± 5.28**
**Ferulic acid**	21.16 ± 3.2	3.17 ± 0.48	6.35 ± 0.96
**Rutin**	17.55 ± 2.9	2.63 ± 0.44	5.27 ± 0.87
**Ellagic acid**	24.89 ± 5.0	3.73 ± 0.75	7.74 ± 1.5
**o-Coumaric acid**	10.72 ± 1.1	1.61 ± 0.17	3.22 ± 0.33
**Resveratrol**	**822.57 ± 13.7**	**123.39 ± 2.1**	**246.77 ± 4.11**
**Total**	**4180.86 ± 18.5**	**627.13 ± 2.78**	**1254.26 ± 5.55**

DPPH, 1,1-diphenyl-2-picrylhydrazyl radical; H_2_O_2_, hydrogen peroxide radical; LDPE, low-dose of date pit extract; HDPE, high-dose of date pit extract.

**Table 3:** The mineral content of DPE.

Mineral Content	DPE (µg/g Extract)	LDPE (150 mg/kg)	HDPE (300 mg/kg)
Ca	44,675.673	6701.35	13,402.7
Cu	1121.442	168.22	336.43
Fe	1488.990	223.35	446.7
K	236,567.019	35,485.053	70,970.11
Mg	37,368.077	5605.21	11,210.42
Mn	133.269	19.99	39.98
Na	35,755.288	5363.29	10,726.59
P	34,262.933	5139.44	10,278.88
Se	20.817	3.12	6.25
Zn	697.163	104.57	209.15

DPE, date pit extract; LDPE, low-dose of date pit extract; HDPE, high-dose of date pit extract.

**Table 4:** Bone pro- and antioxidant parameters.

	MDA Level (µmol/mg Protein)	NO Level (µmol/mg Protein)	GSH Content (µmol/mg Protein)	GPx Activity (U/mg Protein)	GST Activity (u/mg Protein)	SOD Activity (U/mg Protein)
Control	1.9534 ± 0.231	369.22 ± 12.425	10.2953 ± 0.483	58.1272 ± 8.152	0.727 ± 0.041	1.337 ± 0.059
LDPE	1.2168 ± 0.055 *	296.44 ± 50.414 *	10.9006 ± 0.729	54.4612 ± 2.901	0.7637 ± 0.044	1.3448 ± 0.085 *
HDPE	1.063 ± 0.129 *	235.23 ± 12.130 *	13.8351 ± 0.492 *	56.0947 ± 4.179	1.3592 ± 0.044 *	1.6993 ± 0.040 *
DEXA	2.2015 ± 0.033 *	861.26 ± 17.011 *	6.287 ± 0.290 *	23.257 ± 1.874 *	0.389 ± 0.028 *	0.8987 ± 0.057 *
LDPE + DEXA	1.0653 ± 0.074 *^#^	365.8 ± 24.901 ^#^	15.9467 ± 0.646 *^#^	72.4151 ± 4.680 *^#^	1.0359 ± 0.041 *^#^	1.5423 ± 0.062 *^#^
HDPE + DEXA	1.5578 ± 0.084 *^#^	338.29 ± 22.747 ^#^	17.9689 ± 0.637 *^#^	57.4594 ± 3.724 ^#^	1.34 ± 0.067 *^#^	1.4421 ± 0.044 *^#^
IPRI + DEXA	1.1083 ± 0.050 *^#^	441.28 ± 22.444 *^#^	6.608 ± 1.116 *	55.2588 ± 3.020 ^#^	1.0398 ± 0.017 ^#^*	1.336 ± 0.125 ^#^

Values represent the mean ± SD of eight rats/group. * *p* < 0.05 vs. control; # *p* < 0.05 vs. DEXA using ANOVA (one-way) followed by Post Hoc Test (Tukey’s test). LDPE, low-dose of date pit extract (150 mg/kg); HDPE, high-dose of date pit extract (300 mg/kg); DEXA, dexamethasone (1 mg/kg); LPDE + DEXA, low-dose of date pit extract + dexamethasone; HPDE + DEXA, high-dose of date pit extract + dexamethasone; IPRI + DEXA, ipriflavone (50 mg/kg) + dexamethasone; MDA, malondialdehyde; NO, nitric oxide; GSH, reduced glutathione; GPx, glutathione peroxidase; GST, glutathione-S-transferase and SOD superoxide dismutase.

**Table 5:** Histopathological findings in different experimental groups.

	Epiphyseal Plate Thickness (µm)	Trabecular Bone Quality Score *	Trabecular Bone Area Score **	Osteoblast Mean Count/HPF
Control	277	0	0	18
LDPE	233	0	0	16
HDPE	229	0	0	16
DEXA	**80**	**2**	**3**	**4**
LDPE + DEXA	210	0	1	18
HDPE + DEXA	181	1	1	13
IPRI + DEXA	204	1	1	15

* Score 0: normal structure, 1: partially reduced, 2: markedly reduced, and 3: absent trabecular bone. ** Score 0: 90–100%, 1: 60–90%, 2: 30–60%, and 3: <30% of field is trabecular bone area. HPF, high power field; LDPE, low-dose of date pit extract (150 mg/kg); HDPE, high-dose of date pit extract (300 mg/kg); DEXA, dexamethasone (1 mg/kg); LPDE+DEXA, low-dose of date pit extract + dexamethasone; HPDE + DEXA, high-dose of date pit extract + dexamethasone and IPRI + DEXA, ipriflavone (50 mg/kg) + dexamethasone.

## Data Availability

The datasets generated during the current study are available from the corresponding author on reasonable request.
